# Human and economic resources for empowerment and pregnancy-related mental health in the Arab Middle East: a systematic review

**DOI:** 10.1007/s00737-018-0843-0

**Published:** 2018-05-02

**Authors:** Laurie James-Hawkins, Eman Shaltout, Aasli Abdi Nur, Catherine Nasrallah, Yara Qutteina, Hanan F. Abdul Rahim, Monique Hennink, Kathryn M. Yount

**Affiliations:** 1 University of Essex, Colchester, UK; 2 Kingston University, London, UK; 3 Emory University, 1518 Clifton Rd., Atlanta, GA 30329 USA; 4 Palo Alto Medical Foundation Research Institute, Palo Alto, CA USA; 5 KU Leuven, Leuven, Belgium; 6 Qatar University, Doha, Qatar

**Keywords:** Systematic review, Depression, Mental health, Pregnancy, Middle East, Women’s empowerment

## Abstract

This systematic review synthesizes research on the influence of human and economic resources for women’s empowerment on their pre- and postnatal mental health, understudied in the Arab world. We include articles using quantitative methods from PubMed and Web of Science. Two researchers reviewed databases and selected articles, double reviewing 5% of articles designated for inclusion. Twenty-four articles met inclusion criteria. All 24 articles measured depression as an outcome, and three included additional mental health outcomes. Nine of 17 studies found an inverse association between education and depression; two of 12 studies found contradictory associations between employment and depression, and four of six studies found a positive association between financial stress and depression. These results suggest that there is a negative association between education and depression and a positive association between financial stress and depression among women in the Arab world. Firm conclusions warrant caution due to limited studies meeting inclusion criteria and large heterogeneity in mental health scales used, assessment measures, and definitions of human and economic resources for women’s empowerment. It is likely that education reduces depression among postpartum women and that financial stress increases their depression. These findings can be used to aid in the design of interventions to improve mother and child outcomes. However, more research in the Arab world is needed on the relationship between human and economic resources for women’s empowerment and perinatal mental health, and more consistency is needed in how resources and mental health are measured.

Mental health is one of the most neglected public health issues in the Arab world (Haque et al., 2015; Rezaeian, 2010), where there is generally a dearth of rigorous research on mental health conditions among women (Rezaeian, 2010). Medical and public health professionals are concerned about women’s mental health in the perinatal period because common psychiatric disorders, including depression, are more likely to occur then (Satyanarayana et al., 2011). Depression is the most frequently occurring mental health condition among women of childbearing age in low- and middle-income countries (Parsons et al., 2012). Negative consequences have been found for mother and child, including impairments in mother-child interactions and emotional and cognitive disruption of infant development (Haque et al., 2015; Parsons et al., 2012). Poor mental health outcomes among Arab women have been associated with strong patriarchal cultures, which limit opportunities and autonomy for women (Yount and Smith 2012; Yount et al. 2014; Shaikh et al. 2017; Douki et al., 2007).

Women’s empowerment is a critical component of mental health. Thus, women’s empowerment includes the ability or agency to make choices and gain control over available resources to affect better outcomes in their lives (Yount et al. 2015; Campbell and Mannell, 2016; Kabeer, 1999). Resources for empowerment are generally considered to consist of three main categories: (1) financial or economic resources (income, employment, or assets); (2) human resources (education or learned skills); and (3) social resources (social support from family or others; Kabeer, 1999). Gender gaps in mental health may arise from reduced opportunities, status, resources, or power for women in the Arab world (Yount et al. 2014; Hill and Needham, 2013). Thus, human and economic resources for empowerment may influence women’s mental health during and immediately after pregnancy and especially strongly in settings in which women’s access to these resources is constrained (Bener, 2013). Economic resources have been shown to influence women’s empowerment in that they provide the means to exert control over her life by generating income (Kabeer et al., 2013) or increasing her bargaining ability within the home (Kabeer, 2016). Human resources, such as education, are entwined with economic resources in that education and other learned skills can provide the means for women to gain economic resources (Kabeer et al., 2013), often through employment (Hanmer and Klugman, 2016; Kabeer, 2016). Education may also provide women with raised awareness of their own rights and entitlements and allow them to have greater influence over how resources are distributed within the household (Kabeer, 2016).

Researchers have identified possible human and economic risk factors for depression during pregnancy and after delivery (Farr et al., 2014; Scheyer and Urizar Jr., 2016). Those risk factors include low levels of education among women (Fall et al., 2013; Miyake et al., 2012), personal income that is too low to meet basic needs or other financial difficulties (Lancaster et al., 2010; Scheyer and Urizar Jr., 2016), and a lack of engagement in the workforce (Fall et al., 2013; Miyake et al., 2012). While other risk factors such as intimate partner violence and life stressors (Lancaster et al., 2010) have been studied, less attention has been paid to the influence of human and economic resources for women’s empowerment on prenatal and postpartum mental health. Therefore, there is a need for a synthesis of existing research to aid in the design of interventions to improve mother and child outcomes.

In the Arab world, the issues affecting women’s mental health are diverse, reflecting the socioeconomic and cultural diversity of the region. National population policies have tended to focus on family planning services and the reduction of total fertility, rather than the scope and quality of antenatal and postnatal care services (Kronfol, 2012). A recent review of barriers to health care in the Arab world found mental health services to be especially limited in scope, accessibility, and affordability within the public sector (Kronfol, 2012). Women’s empowerment as an influence on mental health is especially important in the Arab world because of well-documented constraints on women’s agency (Kandiyoti, 1988), including reproductive agency or freedom, such as deciding if and when to have a child (Eyadat, 2013). Worldwide, reproductive health services have marginalized mental health (World Health et al., 2009). This is especially true of the Arab world, where mental health services are often inadequate and mental illness is stigmatized (Sewilam et al., 2015). Health providers and policy-makers are just beginning to recognize mental health conditions as contributors to the overall disease burden (Rashad, 2014).

Research on the prevalence of perinatal depressive symptoms, specifically, in the Arab world is scarce, although the onset of new cases of depressive symptoms is most common during the prenatal period (Fuggle et al., 2002). Approximately 10% of women worldwide experience some negative mental health condition during pregnancy, including depressive symptoms (World Health Organization, n.d.). Women who are new mothers also are at higher risk of poor mental health outcomes in general, with 10–15% experiencing postpartum depressive symptoms specifically (Haque et al., 2015; World Health Organization, n.d.). Existing studies, however, have shown that Arab women, living in Arab countries, typically experience higher rates of postpartum depressive symptoms compared to women in other world regions (Chaaya et al., 2002; Green et al., 2006). Given the lack of existing literature on maternal mental health and the known adverse health outcomes for mother and child, there is a clear need for further study on the prevalence of prenatal and postnatal depressive symptoms in the Arab world. This article systematically reviews all studies in the Arab world examining the influence of women’s human or economic resources for empowerment on their mental health in the prenatal and postnatal periods.

## Methods

### Search terms, databases, and search strategies

We used Cochrane Review guidelines (Higgins and Green, 2008) to conduct our literature search. Search terms were identified, piloted, and revised in PubMed and Web of Science databases. Searches were conducted to capture all articles published up to April 1, 2016, the date of the final search conducted. The full search string used to identify potentially relevant articles in each database (Table 1) covered four domains: (1) human and economic resources for women’s empowerment, (2) geographical region, (3) pregnancy/postpartum, and (4) mental health outcomes. The search criteria included peer-reviewed articles in English, French, or Arabic. A title and abstract review was conducted against the inclusion criteria, followed by a full-text review of relevant articles. An ancestry search of references sections was conducted for included articles, and each article’s first/corresponding author was contacted to identify relevant articles in the gray literature.

**Table 1:** Search terms for identifying studies of the effect of human and economic resources for women’s empowerment on mental health during pregnancy in the Arab Middle East

Human and economic resources		Pregnancy		Arab Middle East		Mental health outcomes
Resources						
Material Resources		Pregnancy		Algeria		
Access		Natal		Bahrain		
Ownership		Prenatal		Comoros		
Expenses		Perinatal		Djibouti		
Expenditures		Postnatal		Egypt		
Assets		Gestation		Iraq		
Wealth		Expecting		Jordan		
Possession		Mother		KSA		
Welfare		Pregnant		Kuwait		
Economic Security		Parity		Lebanon		
Savings		Gravidity		Libya		
Employ*		Antenatal		Mauritania		Health
Income		Labor		Morocco		Psychological Well-Being
Occupation		Birth		Oman		Mental Well-Being
Socioeconomic Status	AND	Childbirth	AND	Qatar	AND	Mental Illness
Financ*		Matern*		Saudi Arabia		Depression
Residence		Neonatal		Somalia		Depressive Symptoms
Women’s Agency		Fetal		Sudan		Anxiety
Agency		Baby		Syria		Stress
Women’s Empowerment		Delivery		Tunisia		
Empowerment		Child Bearing		UAE		
Women’s Decision-making		Parturient		United Arab Emirates		
Decision-making		Obstetric Care		West Bank		
Women’s Mobility		Cesarean Section		Gaze		
Mobility		With Child		Palestine		
Women’s Autonomy		Enceinte		Yemen		
Women’s Freedom of Movement		Conception		Middle East		
Freedom of Movement		Impregnate		Arab World		
Gender Equality		Conceive		MENA		
Women’s Status				North Africa		
Status						

### Selection of studies

Our inclusion and exclusion criteria were established a priori and refined during an extensive pilot phase (Table 2). We excluded studies if neither human nor economic resources were included, if the study population did not consist of Arab pregnant or postpartum women living in an Arab country, or if no mental health outcome was measured.^1^ We defined the postpartum period as up to 1 year after birth (Canadian Mental Health Association, 2017). We were interested in the relationship between human and economic resources for empowerment and perinatal mental health in women experiencing a “typical” pregnancy and as such included mental health outcomes that were defined as “normal” by the Diagnostic Statistical Manual of Psychiatric disorders (DSM) such as depression and anxiety. We excluded studies including only women who experienced life-threatening conditions or extreme psychopathology defined as mental health problems or psychological stressors that are classified as “abnormal” by the DSM such as schizophrenia. Systematic reviews and studies using qualitative methods also were excluded. All studies published before March 2016 were included.

**Table 2:** Final inclusion and exclusion criteria

Criteria	Included	Excluded	Rationale
Sampling method	Population-based, and clinic-based except those admitted for psychopathology or serious pregnancy complications	Convenience-based, clinic-based if sample admitted for psychopathology or serious pregnancy complications	The study aims to understand the effect of empowerment on common pregnancy-related mental health outcomes, rather than mental health complicated by medical concerns or psychiatric disorders (e.g., schizophrenia or mania).
Analysis	Bivariate analysis; quantitative analysis	Anything less than bivariate analysis; qualitative analysis	Bivariate analysis is included as a minimum in order to understand the complex relationship between dimensions of empowerment and mental health in pregnancy.
Date	All dates were included	No elimination based on date	Studies based in any time period would contribute to the objectives of this study. Given the limited research available on mental health among pregnant women within the geographic region of interest, articles were not excluded based on the date of publication.
Geographic region	Arab world (as defined by the Arab League and World Bank)	Non-Arab countries; Arab populations outside Arab countries (e.g., refugees)	The focus of this review is on perinatal mental health in women from Arab countries, as these countries have seen a dramatic improvement in maternal health and child survival over the past few decades as a result of better living standards and improvements in health care services.
Population of interest	Arab pregnant women and/or women in the perinatal period (22 weeks of gestation to 7 days after birth; WHO, 2016) and postnatal period (up to 1 year after the birth of a child).	More than 1 year after the birth of a child	The period of time specified represents the focus of interest for the purposes of the review.
Outcome variable	Mental and psychological health or well-being; depressive symptoms; anxiety symptoms; perceived psychological stressors	Psychopathology and/or psychiatric disorders	This review is concerned with common mental health problems/psychological stresso rs that are not classified as “abnormal” by the Diagnostic Statistical Manual of Psychiat ric disorders (DSM; “abnormal” mental health problems include problems such as sch izophrenia or bipolar disorder), and symptoms that have not progressed to mental diso rders (e.g., we are looking at anxiety symptoms not generalized anxiety disorder).
Exposure variable	Agency and/or resources for empowerment (or disempowerment; e.g., domestic violence)	Any items that did not fall within the conceptual framework of empowerment outlined by Kabeer (1999)	The definition of empowerment used in this review is based on Kabeer’s (1999) frame work, which includes resources and agency. Different terms were also used to describ e these analogous constructs (e.g. decision-making or wealth).
Language	English, Arabic, and French	All other languages unless translation was provided	Majority of the published literature in this field is in English. Some studies conducted in Tunisia and Morocco were published in French journals and were reviewed by French speaking researchers.
Peer reviewed	Peer reviewed	Non-peer reviewed	The use of peer-reviewed articles reflects this review’s focus on using the highest-quality research .

### Data extraction and analysis

**Fig. 1:**
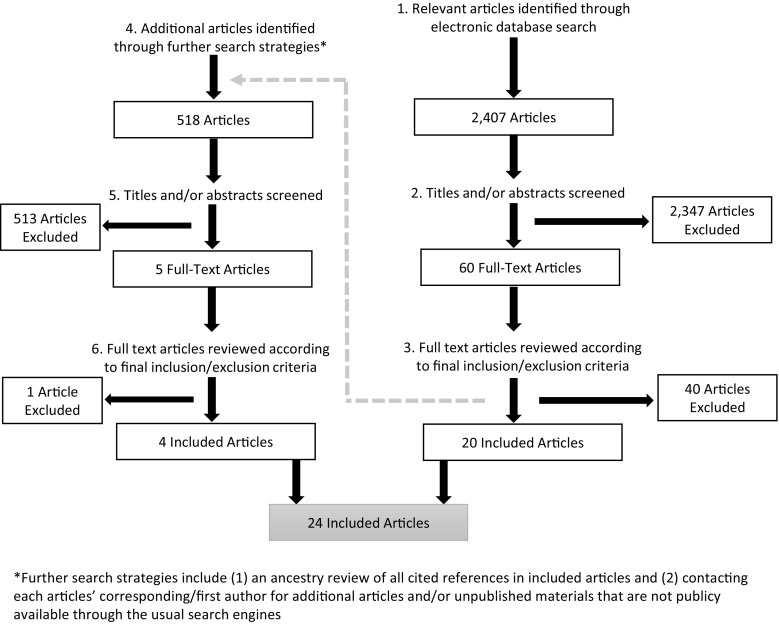
Steps in the search, screening, and selection of studies

A total of 2407 articles were identified and screened for further review based on the titles and abstracts (Fig. 1). We excluded 2347 articles because they did not address the constructs or population of interest. The remaining 60 articles underwent a full-text review by one of three researchers (ES, AN, CN) resulting in 20 articles that met all inclusion criteria. Articles were excluded if they did not contain a measure of human or economic resources or if those measures were not used in an analysis with mental health as the outcome. Reference and key author searches identified five additional articles. Four met final inclusion criteria after a full-text review, for a total of 24 included articles. The Cochrane Review data extraction form was adapted for use with cross-sectional and observational studies (Norwegian Knowledge Centre for the Health Services, 2013). Five percent of included articles underwent a second review for data extraction to ensure consistency.

### Assessment of study quality and validity

We used the STrengthening the Reporting of OBservational Studies in Epidemiology (STROBE) checklist to assess study quality (Von Elm et al., 2007). The STROBE checklist uses 22 criteria, so each article was assigned a score between 1 and 22. Articles were rated on quality by one researcher (LJH). Articles with scores of 14 or below were designated as “low quality,” scores between 15 and 17 were “medium quality,” and scores of 18 and above were “high quality” (James-Hawkins et al. 2016; Table 3). A sub-sample of articles were double scored by a second researcher (YQ) for consistency. The lowest quality articles generally did not define outcomes and exposures adequately, explain how variables were handled in the analysis, report sample attrition, discuss potential sources of bias, explain missing data adequately, provide their source of funding, or discuss generalizability of their results. Medium-quality articles generally did not address potential bias, explain missing data, or provide their funding source. High-quality studies generally did not explain how missing data were addressed.

**Table 3:** Characteristics of included studies (*N* = 24)

Author	Year	Sample type	Sample design	Pregnancy/postpartum period	N	Country	Study Quality
Abdelhai and Mosleh	2015	Probability sample	Systematic sampling	First trimester/second trimester/third trimester	376	Egypt	16 Medium
Abuidhail and Abujilban	2014	Non-probability sample	Convenience sampling	Third trimester	218	Jordan	12 Low
Abujilban et al.	2014	Non-probability sample	Convenience sampling	Third trimester	218	Jordan	15 Medium
Agoub, Moussaoui, and Battas	2005	Non-probability sample	Convenience sampling	Postpartum	144	Morocco	12 Low
Al-Azri et al.	2016	Probability sample	Systematic sampling	First trimester/second trimester/third trimester	959	Oman	16 Medium
Al Dallal and Grant	2012	Non-probability sample	Convenience sampling	Postpartum	237	Bahrain	14 Medium
Alharbi and Abdulghani	2014	Non-probability sample	Convenience sampling	Postpartum	352	Saudi Arabia	14 Medium
Al Hanai and Al Hanai	2014	Non-probability sample	Convenience sampling	Postpartum	282	Oman	12 Low
Bener	2013	Probability sample	Systematic sampling	Postpartum	1659	Qatar	16 Medium
Bener, Burgut et al.	2012	Non-probability sample	Convenience sampling	Postpartum	1379	Qatar	17 Medium
Bener, Gerber, and Sheikh	2012	Probability sample	Systematic sampling	Postpartum	1659	Qatar	19 Medium
Burgut, Bener, Ghuloum, and Sheikh	2013	Non-probability sample	Convenience sampling	Postpartum	1379	Qatar	16 Medium
Chaaya et al.	2002	Non-probability sample	Convenience sampling	Postpartum	396	Lebanon	17 Medium
El-Khoury, Karam, and Melham	1999	Non-probability sample	Convenience sampling	Postpartum	150	Lebanon	15 Medium
Green, Broome, and Mirabella	2006	Non-probability sample	Convenience sampling	Postpartum	125	United Arab Emirates	15 Medium
Hamdan and Tamim	2011	Non-probability sample	Convenience sampling	Second trimester/third trimester/postpartum	137	United Arab Emirates	19 High
Khabour et al.	2013	Probability sample	Stratified Sampling	Postpartum	370	Jordan	16 Medium
Lteif, Kesrouani, and Richa	2005	Non-probability sample	Convenience sampling	First trimester, second trimester, third trimester	79	Lebanon	16 Medium
Masmoudi et al.	2014	Non-probability sample	Convenience sampling	Postpartum	213	Tunisia	17 Medium
Masmoudi et al.	2010	Non-probability sample	Convenience sampling	Postpartum	301	Tunisia	16 Medium
Masmoudi et al.	2008	Non-probability sample	Convenience sampling	Postpartum	213	Tunisia	15 Medium
McHichi Alami, Kadri, and Berrada	2006	Non-probability sample	Convenience sampling	First trimester/second trimester/third trimester/postpartum	100	Morocco	10 Low
Mohammad et al.	2011	Non-probability sample	Convenience sampling	First trimester/second trimester/third trimester/postpartum	353	Jordan	15 Medium
Moh’d Yehia, Callister, and Hamdan-Mansour	2013	Non-probability Sample	Convenience sampling	Postpartum	300	Jordan	17 Medium

All studies used primary data collection and were clinic based

We assessed each study for bias in terms of selection, measurement error, statistical analysis, and confounders using a previously adapted tool (Yount and Smith 2012). One reviewer (LJH) classified each study as low, medium, or high risk for each category. We evaluated selection bias based on sampling design and response rates. Non-probability samples were considered high risk and probability samples were considered low risk. Response rates of 80% or higher were low risk, 60–80% were moderate risk, and below 60% or an unreported response rate was considered high risk. If a study was low in one and moderate or high in the other, it was classified as moderate risk. We assessed measurement error based on reporting of measures of internal consistency (e.g., Cronbach’s alpha or similar). Measurement error was rated as low risk if the study authors addressed the reliability of all key measure(s) (mental health, and human/economic resources), moderate risk if they addressed reliability of mental health or resources, and high risk if reliability of neither was addressed. Statistical analysis bias was assessed based on statistical tests used. Studies with clear descriptions of the analysis plan and statistical tests used were low risk. Studies lacking clear information were considered as moderate risk. High risk studies did not report the statistical tests used. Finally, confounder risk was assessed based on the inclusion of confounders in the analysis. Low risk studies included a comprehensive set of confounders, moderate risk studies included minimal confounders, and high risk studies included no confounders (Table 4).

**Table 4:** Assessment of potential threats to study validity (*N* = 24)

Article	Selection bias	Measurement error	Statistical analysis bias	Confounder bias
Abdelhai and Mosleh, 2015	Low risk	Moderate risk	Low risk	Low risk
Abuidhail and Abujilban, 2014	Moderate risk	Moderate risk	Low risk	High risk
Abujilban et al., 2014	High risk	Low risk	Low risk	Moderate risk
Agoub et al., 2005	Moderate risk	Low risk	Low risk	High risk
Al-Azri et al., 2016	Low risk	Moderate risk	Low risk	Low risk
Al Dallal and Grant, 2012	High risk	Low risk	Low risk	High risk
Alharbi and Abdulghani, 2014	High risk	High risk	Moderate risk	Moderate risk
Al Hinai and Al Hinai, 2014	High risk	High risk	Low risk	Moderate risk
Bener, 2013	Moderate risk	High risk	Low risk	Low risk
Bener, Burgut et al., 2012	Moderate risk	High risk	Low risk	Moderate risk
Bener, Burgut et al., 2012	High risk	High risk	Low risk	Low risk
Burgut et al., 2013	Moderate risk	High risk	Low risk	Low risk
Chaaya et al., 2002	High risk	High risk	Low risk	Low risk
El-Khoury et al., 1999	High risk	High risk	Moderate risk	High risk
Green et al., 2006	High risk	High risk	High risk	High risk
Hamdan and Tamim, 2011	High risk	Moderate risk	Low risk	High risk
Khabour et al., 2013	Moderate risk	Low risk	Low risk	High risk
Lteif et al., 2005	High risk	High risk	Low risk	High risk
Masmoudi et al., 2014	High risk	High risk	Low risk	High risk
Masmoudi et al., 2008	High risk	High risk	Low risk	High risk
Masmoudi et al., 2010	High risk	High risk	Low risk	High risk
McHichi Alami et al., 2006	High risk	High risk	Low risk	High risk
Mohammad et al., 2011	High risk	Low risk	Low risk	High risk
Moh'd Yehia et al., 2013	High risk	Low risk	Low risk	Moderate risk

## Results

### Characteristics of studies

A total of 24 articles met the inclusion criteria (Table 3). Twenty studies were in English, four in French, and none in Arabic. The studies were published between 1999 and 2016 and conducted in ten different countries. Most studies (*N* = 19) used convenience sampling, with sample sizes ranging from 79 to 1659 women, and an average of 483 participants across studies. Sixteen studies focused on the postpartum period, five on the prenatal period, and three on both. Ten studies included human or economic resources as control variables, and we used data to calculate population proportions for comparison purposes. All studies collected primary data in clinics.

#### Measurement of mental health

The majority of studies (*N* = 21) addressed depression as the sole outcome. Two also included anxiety, and one included anxiety and psychological stress in addition to depression. The Edinburgh Post-Natal Depression Scale (EPDS) was used most commonly (*N* = 16). The Mini International Neuropsychiatric Interview (MINI; *N* = 3), Depression Anxiety Stress Scale (DASS-21; *N* = 2), Beck Depression Inventory (BDI; *N* = 1), Hospital Anxiety and Depression Scale Questionnaire (HADS; *N* = 1), and Depression Detailed Inventory (DDI; *N* = 1) also were used.^2^ How depression was determined varied with researchers using different cut-points to indicate “major depression,” even when using the same scale.

#### Measurement of human and economic resources for women’s empowerment

Human resources were represented by measures of education (*N* = 19), economic resources by measures of employment (*N* = 18), and financial resources (*N* = 17). Education was operationalized in multiple ways, including literate versus not (*N* = 3), ordinal school levels (*N* = 2), or dummy variables such as completed secondary school or more versus less (*N* = 9), and greater than secondary versus secondary or less (*N* = 3). Cut-offs used for education were unclear in three studies. Employment was operationalized as problems at work versus none, among those working (*N* = 2), or working versus not working (*N* = 10). Operationalization of financial resources also varied with three studies using perceptions of financial distress, two using a salary cut-off in local currency, one using a continuous income measure, and one using income satisfaction.

#### Threats to study validity

The majority of studies were rated as high risk on selection bias because they used convenience samples or did not report response rates. Risk of measurement error also was high for most studies. While authors indicated that instruments had been validated in-country, they rarely reported assessments using their sample. Almost all studies presented a description of the statistics and methods used and were rated as low risk for statistical bias. Risk for confounder bias was high overall as there was a general lack of inclusion of confounders.

### Prenatal relationships

#### Education

Four studies assessed the association between education and depressive symptoms (EPDS = 3, MINI = 1). Two studies were medium quality and two were low quality. Two studies using the EPDS found opposite relationships (one positive and one negative), while the third found no relationship. The study using the MINI also found no relationship (Table 5). Thus, the association between education and depression among pregnant women was contradictory and inconclusive.

**Table 5:** The association between pre- and postpartum women’s human and economic resources for empowerment and mental health outcomes in the Arab world

Author	Resource for empowerment	Natal period	Mental health area	Mental health instrument	Break on mental health scale	Measurement metric	Statistical test	Association	Coefficients	Study quality
Abuidhail and Abujilban	Education	Prenatal	Depression	EPDS	Score of ≥ 13 on EPDS vs. < 13	Low education vs. high education (ref.)	Bivariate, means comparison	Positive	*t* = 5.10, *p* = 0.00	12 low
Abujilban et al.	Education	Prenatal	Depression	EPDS	Continuous Measure	Elementary to MA, literate women only	Multivariate, regression	Negative	*B* = − 2.2, *p* < 0.05	15 medium
Al-Azri et al.	Education	Prenatal	Depression	EPDS	Score of ≥ 13 vs. < 13	Secondary or less vs. university	Bivariate, chi-square	None	*χ*^2^ = 2.32, *p* = 0.13	16 medium
McHichi Alami, Kadri, and Berrada	Education	Prenatal	Depression	MINI	Not stated	Illiterate vs. literate (ref.)	Bivariate, logistic regression	None	PPC: *Z* = 0.09, *p* = 0.93	10 low
Al-Azri et al.	Employment	Prenatal	Depression	EPDS	Score of ≥ 13 vs. < 13	Housewife vs. employed	Bivariate, chi-square	None	*χ*^2^ = 0.71, *p* = 0.39	16 medium
Lteif, Kesrouani, and Richa	Employment	Prenatal	Depression	BDI	Score of < 10, 10–18, > 18	Problems at work vs. not (ref.) (only among those working)	Bivariate, logistic regression	Positive	UaOR = 55.8, *p* = 0.001	16 medium
McHichi Alami, Kadri, and Berrada	Employment	Prenatal	Depression	MINI	Not stated	Not working vs. working (ref.)	Bivariate, logistic regression	None	PPC: *Z* = − 0.41, *p* = 0.68	10 low
Al-Azri et al.	Financial	Prenatal	Depression	EPDS	Score of ≥ 13 vs. < 13	< 500 vs. 500–1000 vs. > 1000 Omani Riyals	Bivariate, chi-square	None	*χ*^2^ = 5.01, *p* = 0.08	16 medium
Mohammad et al., 2011	Financial	Prenatal	Depression	EPDS	Score of < 13 on EPDS vs. ≥ 13	Worry about financial problems	Multivariate, regression	Positive	*B* = 0.08, *p* = 0.01	15 medium
Abdelhai and Mosleh	Financial	Prenatal	Depression and anxiety	HADS	Experiencing anxiety and depression vs. neither	Perceived financial distress, 5-point Likert scale	Multivariate, logistic regression	None	UaOR = 1.59, *p* = 0.15 (ref: no perceived financial distress)	16 medium
Bener	Education	Postnatal	Depression, anxiety, and stress	DASS-21	≥ 10 depression ≥ 8 anxiety ≥ 15 stress	≥ Secondary vs. < secondary (ref.)	Bivariate, means comparison	Negative	PPC: *Z* = − 2.70, *p* = 0.01	16 medium
Bener, Gerber, and Sheikh	Education	Postnatal	Stress	DASS-21	≥ 15 stress	< Secondary vs. ≥ secondary (ref.)	Multivariate, logistic regression	Positive	aOR = 1.50, *p* = 0.04	19 high
Bener, Gerber, and Sheikh	Education	Postnatal	Depression	DASS-21	≥ 10 depression	< Secondary vs. ≥ secondary (ref.)	Multivariate, logistic regression	Positive	aOR = 1.50, *p* = 0.01	19 high
El-Khoury, Karam, and Melham	Education	Postnatal	Depression	DDI	Major depression vs. not (cut-offs not reported)	≥ Secondary vs. < secondary (ref.)	Bivariate, means comparison	None	*X*^2^ = 0.18, *p* = 0.67	15 medium
Al Dallal and Grant	Education	Postnatal	Depression	EPDS	Score of ≥ 12 on EPDS vs. < 12	≥ Secondary vs. < secondary (ref.)	Bivariate, means comparison	None	PPC: *Z* = − 1.52, *p* = 0.13	14 medium
Alharbi and Abdulghani	Education	Postnatal	Depression	EPDS	Score of ≥ 10 vs. < 10	≥ Secondary vs. < secondary (ref.)	Bivariate, chi-square	None	*X*^2^ = 0.07, *p* = 0.79	14 medium
Bener, Burgut et al.	Education	Postnatal	Depression	EPDS	Score of ≥ 12 on EPDS vs. < 12	≥ Secondary vs. < secondary (ref.)	Bivariate, means comparison	Negative	PPC: *Z* = − 4.49, *p* = 0.00	17 medium
Burgut, Bener, Ghuloum, and Sheikh	Education	Postnatal	Depression	EPDS	Score of ≥ 12 on EPDS vs. < 12	< Secondary vs. ≥ secondary (ref.)	Multivariate, logistic regression	None	Qatari: aOR = 1.62, *p* = 0.08; Arab non-Qatari: aOR = 0.78, *p* = 0.32	16 medium
Chaaya et al.	Education	Postnatal	Depression	EPDS	Above threshold 12/13 vs. below	Low and high vs. medium (cut-offs not reported, ref. = medium)	Multivariate, logistic regression	None	low: OR 1.12, *p* = 0.77; high: OR 1.98, *p* = 0.20;	17 medium
Green, Broome, and Mirabella	Education	Postnatal	Depression	EPDS	Score of 0–9, 10–12, and ≥ 13 on EPDS	Not reported	Bivariate, means comparison	None	No association, *p* > 0.05	15 medium
Khabour et al.	Education	Postnatal	Depression	EPDS	Score of > 13 on EPDS vs. ≤ 13	≥ Secondary vs. < secondary (ref.)	Bivariate, means comparison	None	PPC: *Z* = 0.99, *p* = 0.32	16 medium
Masmoudi, Charfeddine et al.	Education	Postnatal	Depression	EPDS	Score of > 10 on EPDS vs. ≤ 10	< Secondary vs. ≥ secondary (ref.)	Bivariate, means comparison	Positive	*Z* = 1.04, *p* = 0.30 (PPD); *Z* = 2.80, *p* < 0.01 (intense PPD)	17 medium
Masmoudi, Trabelsi … Jaoua et al.	Education	Postnatal	Depression	EPDS	Score of ≥ 10 on EPDS vs. < 10	≥ Secondary vs. < secondary (ref.)	Bivariate, means comparison	None	*Z* = 0.31, *p* = 0.76	15 medium
Masmoudi, Trabelsi… Hantouche et al.	Education	Postnatal	Depression	EPDS	Score of > 10 on EPDS vs. ≤ 10	Primary, secondary, post-secondary	Bivariate, chi-square	Negative	*X*^2^ = 6.68, *p* = 0.03	16 medium
Agoub, Moussaoui, and Battas	Education	Postnatal	Depression	MINI	MINI case vs. not (cut-offs not reported)	Literate vs. illiterate (ref.)	Bivariate, means comparison	None	PPC: *Z* = 0.22, *p* = 0.83	12 low
McHichi Alami, Kadri, and Berrada	Education	Postnatal	Depression	MINI	Not stated	Illiterate vs. literate (ref.)	Bivariate, means comparison	None	PPC: *Z* = 0.13, *p* = 0.90	10 low
Hamdan and Tamim	Education	Postnatal	Depression	MINI	MINI case vs. not (cut-offs not reported)	> Secondary vs. ≤ secondary (ref.)	Bivariate, means comparison	Negative	PPC: *Z* = 2.08, *p* = 0.04	19 high
Bener, Gerber, and Sheikh	Employment	Postnatal	Depression	DASS-21	≥ 10 depression	Not working vs. working (ref.)	Multivariate, logistic regression	Negative	aOR = 1.6, *p* = 0.00	19 high
Al Dallal and Grant	Employment	Postnatal	Depression	EPDS	Score of ≥ 12 on EPDS vs. < 12	Not working vs. working (ref.)	Bivariate, means comparison	None	PPC: *Z* = 1.34, *p* = 0.18	14 medium
Alharbi and Abdulghani	Employment	Postnatal	Depression	EPDS	Score of ≥ 10 on EPDS vs. < 10	Work or school vs. housewife	Bivariate, chi-square	None	*X*^2^ = 1.73, *p* = 0.19	14 medium
Al Hanai and Al Hanai	Employment	Postnatal	Depression	EPDS	Score of 0–9, 10–12, and ≥ 13 on EPDS	Work difficulties vs. none (ref.) (among working women)	Bivariate, logistic regression	None	At 2 weeks: UaOR 2.41, *p* = 0.01; At 8 weeks UaOR 2.27, *p* = 0.02;	12 low
Bener, Burgut et al.	Employment	Postnatal	Depression	EPDS	Score of ≥ 12 on EPDS vs. < 12	Not working vs. Working (ref.)	Bivariate, Means comparison	None	PPC: *Z* = 1.54, *p* = 0.12	17 medium
Burgut, Bener, Ghuloum, and Sheikh	Employment	Postnatal	Depression	EPDS	Score of ≥ 12 on EPDS vs. < 12	Working vs. not	Multivariate, logistic regression	None	Qatari aOR = 1.78, *p* = n.s. Arab non-Qatari aOR = 0.13, *p* = n.s.	16 medium
Chaaya et al.	Employment	Postnatal	Depression	EPDS	Above threshold 12/13 vs. below	Working vs. not (ref.)	Multivariate, logistic regression	None	uaOR = 0.74, *p* = 0.60;	17 medium
Green, Broome, and Mirabella	Employment	Postnatal	Depression	EPDS	Score of 0–9, 10–12, and ≥ 13 on EPDS	Working vs. not	Bivariate, means comparison	None	No Association, *p* > 0.05	15 medium
Khabour et al.	Employment	Postnatal	Depression	EPDS	Score of > 13 on EPDS vs. ≤ 13	Not working vs. working (ref.)	Bivariate, means comparison	None	PPC: *Z* = − 0.25, *p* = 0.80	16 medium
Agoub, Moussaoui, and Battas	Employment	Postnatal	Depression	MINI	MINI case vs. Not (cut-offs not reported)	Working vs. not (ref.)	Bivariate, means comparison	None	PPC: *Z* = 0.00, *p* = 1.0	12 low
McHichi Alami, Kadri, and Berrada	Employment	Postnatal	Depression	MINI	Not stated	Working vs. not (ref.)	Bivariate, means comparison	None	PPC: *Z* = − 1.28, *p* = 0.20	10 low
Hamdan and Tamim	Employment	Postnatal	Depression	MINI	MINI case vs. not (cut-offs not reported)	Working vs. not (ref.)	Bivariate, means comparison	None	PPC: *Z* = 0.95, *p* = 0.34	19 high
Bener, Burgut et al.	Financial	Postnatal	Depression	EPDS	Score of ≥ 12 on EPDS vs. < 12	Difficulty managing income vs. not (ref.)	Multivariate, logistic regression	Positive	aOR = 2.37, *p* < 0.001	17 medium
Khabour et al.	Financial	Postnatal	Depression	EPDS	Score of > 13 on EPDS vs. ≤ 13	Not satisfied with income vs. satisfied (ref.)	Bivariate, means comparison	Positive	*Z* = 2.17, *p* = 0.03	16 medium
Moh’d Yehia, Callister, and Hamdan-Mansour	Financial	Postnatal	Depression	EPDS	Continuous Measure	Monthly income	Multivariate, regression	Negative	*B* = − 0.54, *p* = 0.03	17 high
McHichi Alami, Kadri, and Berrada	Financial	Postnatal	Depression	MINI	Not stated	Financial distress vs. none (ref.)	Bivariate, means comparison	None	PPC: *Z* = − 0.67, *p* = 0.50	10 low

*EPDS* Edinburgh Postnatal Depression Scale, *MINI* Mini International Neuropsychiatric Interview, *BDI* Beck Depression Inventory, *HADS* Hospital Anxiety and Depression Scale, *DASS*-*21* Depression Anxiety Stress Scale (21 item version), *DDI* Depression Detailed Inventory

; PPC=population proportion comparison; UaOR=Unadjusted odds ratio; aOR=Adjusted odds ratio

#### Employment

Three studies examined the relationship between women’s employment status and prenatal depressive symptoms. One study used the EPDS and found no relationship. One study used the BDI and found a positive relationship among women who were employed and reported problems at work. The third study used the MINI to compare working versus nonworking women and found no association. Two studies were medium quality, and the other was low quality (Table 5). Overall, two out of three studies found no relationship between employment and depression among pregnant women.

#### Financial stress

Three studies examined financial stress and prenatal mental health. Two of the studies used the EPDS with one finding a significant positive association and the other finding no association. The third study used the HADS and found no relationship. All three studies were of medium quality (Table 5). Overall, two out of three studies found no association between financial stress and depression among pregnant women.

### Postnatal relationships

#### Education

Sixteen studies examined the relationship of schooling attainment with depression, anxiety, or psychological stress (EPDS = 10, MINI = 3, DASS-21 = 2, DDI = 1). Among studies using the EPDS, six different cut-offs were used to determine if depression was present, and eight different metrics were used to measure schooling attainment. All studies using the EPDS were of medium quality. Two medium-quality studies used the DASS-21 to assess depressive symptoms, both finding a negative relationship. Two other studies examined the relationship between schooling attainment and postnatal depressive symptoms using the MINI instrument to assess depressive symptoms, with neither finding a relationship. Overall, five the 16 studies found a negative relationship between schooling attainment and depression, while 11 studies found no association. However, this result appears to be at least somewhat instrument dependent. Overall, one study was high quality, 12 studies were medium quality, and two were low quality (Table 5). All studies that found an association were of medium or high quality, suggesting that there may be a negative association between education and depression for postpartum women.

#### Employment

Twelve studies examined the association between employment and postnatal depression. Seven of these studies compared women who were working with women who were not working, while one compared trouble at work among women working. Eight studies used the EPDS and found no association between women’s work status and postnatal depression. Three additional studies used the MINI and also found no association. One high-quality study using the DASS-21 found lower depression among women who were not working. Of the other 11 studies, one was of high quality, seven were medium quality, and three were low quality (Table 5). Overall, no association was found between employment status and postpartum depression.

#### Financial stress

Four studies examined financial issues and mental health in the postnatal period. Three of these used the EPDS and found an inverse association between actual income or satisfaction with income and levels of depressive symptoms. Of these, one study was high quality and two were medium quality. One low-quality study used the MINI and found no association (Table 5). Overall, an association between financial stress and postpartum depression is plausible.

## Discussion

This systematic review examined how women’s human and economic resources for empowerment were associated with their perinatal mental health. Overall, no association is apparent in the prenatal period. However, we cautiously conclude that there is evidence for a negative relationship between schooling and postnatal depression. However, this may be dependent on the instrument used to assess depression, and the low number of studies addressing this relationship makes it difficult to draw strong conclusions. Few studies addressed women’s financial stress and postnatal mental health and they used a variety of instruments and populations; however, the available evidence suggests a positive relationship between financial stress and negative mental health outcomes. Almost none of the studies examining employment and postnatal mental health found an association. While there were substantially more studies examining women’s resources for empowerment and postnatal mental health than for prenatal mental health, multiple factors varied across these studies such as (1) the instrument used to measure depression, (2) how depressive symptoms were assessed, and (3) the metrics used to measure the resource. Thus, conclusions drawn must be tempered by the knowledge that both operationalization and measurement error are likely to have impacted the results we considered.

The tentative negative association between schooling attainment and depression in the postnatal period is important because of the strong emphasis on marriage and family for women living in the Arab world (Barakat, 2005). In many countries in the Arab world, women marry before completing their secondary schooling and do not have the opportunity to continue their education. At the same time, women are considered the “mothers of the nation” and tasked with transmission of culture to younger generations (p. 45, Kandiyoti, 1991). When women marry young, they often do not have strong identities outside their role as wife and mother (Barakat, 2005; Kandiyoti, 1988). The lack of schooling attainment and relative isolation of these women is likely to contribute to depression, as tentatively suggested by the findings of this review.

High financial stress has been associated with pre- and postpartum depression among women in many regions of the world (Eastwood et al., 2011; Ehrlich et al., 2010; Yelland et al., 2010). The tentative association found here suggests that this relationship may be true for women in the Arab world, as well. Women experiencing financial stress may be overwhelmed by trying to care for their child and attend to household duties. In addition, women in the Arab world may be restricted from working by their husbands and thus may be unable to contribute financially to their family or may not have control over any financial resources (James-Hawkins et al. 2016). The inability to either contribute to the household financially or to control resources may leave women feeling overwhelmed and helpless. However, employment status may not be as relevant as type of job or the conditions of employment, which were not detailed in the studies reviewed. Also, the work-related options available to women may not be desirable enough for women to pursue work, and so the alternative of staying home is preferred.

A limitation of the articles reviewed is the lack of theory about how women’s resources for empowerment may influence their perinatal mental health. This concept has been shown to be relevant to mental health, with researchers finding significant associations between women’s empowerment and levels of anxiety in women of reproductive age in the Arab world (Yount and Smith 2012; Yount et al. 2014). The small number of studies identified also presents a problem. Given the demonstrated importance of mental health in the pre- and postnatal periods for positive mother and child outcomes (Glover, 2014; Leis et al., 2014), and the impact of reduced human and economic resources on women’s health and well-being in the Arab world (Haghighat, 2013, 2014; Price, 2016), more studies should be conducted on this topic to allow for more robust conclusions. Focus on a wider scope of mental health outcomes is also needed. While other mental health issues in pregnancy such as anxiety, mood, and psychological stress have been well-studied in Western countries (Hall et al., 2014, 2015; Redshaw and Henderson, 2016; Rubertsson et al., 2014), they have been virtually ignored in the Arab world. A critical limitation of these studies was the inconsistency in how scales were used and how outcomes were operationalized. We encourage researchers interested in perinatal mental health outcomes to spend time creating standards for the use of different scales and suggest they validate those standards in relevant, culturally appropriate contexts as a part of the research process.

Future research should expand the number of psychological conditions assessed in this region, as the current literature focuses almost exclusively on depression, ignoring other mental health issues that may influence the health of the mother and child such as stress, anxiety, or negative mood states (Hall et al., 2014, 2015; Redshaw and Henderson, 2016; Rubertsson et al., 2014). Overall, the pattern of associations found appeared to be attributable to the variety of instruments used, how depression was assessed, and how women’s human and economic resources for empowerment were measured, which speaks more to the consistency and quality of measurement in the literature than to any substantive conclusions about the actual association between domains. This lack of uniformity in the use of consistent cut-off scores was especially true for the EPDS, the most frequently used instrument. Differences in how depression was determined may be complicated by the wide variety of ways in which human and economic resources for empowerment were measured. Finally, the research conducted spanned ten different countries in the MENA region. Overall, there was limited research in the Arab world that addressed mental health.

### Limitations and strengths

First, this systematic review is limited to the examination of common and usually less severe mental health outcomes and does not address severe pathologies such as schizophrenia or bipolar disorder. Thus, it may be that there are associations between the human and economic resources for empowerment assessed and other psychological morbidities. However, we intentionally focused on less severe mental pathology in order to assess symptoms of mental distress that are more likely to be experienced by women in the region. Second, there may be older articles that were not included in the electronic databases we searched. However, it is likely that older research has been included in the databses at least in citation form and thus would have been identified. Third, it is possible that our selection of databases missed relevant literature for this review. However, extensive piloting of other databases suggested that we would not gain from including them. A major strength of this review is that it is the first systematic review of mental health issues experienced during pregnancy and in the postpartum periods in the Arab world.

#### Implications for policy and practice

We identified possible associations between schooling attainment and depression and between financial stress and depression. These findings suggest that closer attention should be paid to women’s access to enabling resources when evaluating them for mental health issues during or after pregnancy. Overall, further research is needed on mental health and the perinatal period both to supplement the existing limited research and to clarify relationships tentatively identified here. Also, we suggest that the international community of researchers attempt to determine specific ways in which human and economic resources for women’s empowerment can be measured in more standardized ways.

## Footnotes

1 The majority of the articles rejected during the title and abstract review were excluded for being systematic reviews, having a non-human study population, studying non-pregnant women or non-Arab samples, not measuring a mental health outcome, or the mental health outcome was a severe psychopathology. The remaining articles were then subjected to a full-text review and articles were excluded because economic and human resources for women’s empowerment were not analyzed in relation to mental health.

2 One study used two different scales for measurement of depression.

## References

[CR1] Abdelhai R, Mosleh H (2015). Screening for antepartum anxiety and depression and their association with domestic violence among Egyptian pregnant women. J Egypt Public Health Assoc.

[CR2] Abuidhail J, Abujilban S (2014). Characteristics of Jordanian depressed pregnanct women: a comparison study. J Psychiatr Ment Health Nurs.

[CR3] Abujilban SA, Abuidhail J, Al-Modallal H, Hamaideh S, Mosemli O (2014). Predictors of antenatal depression among pregnant women in their third trimester. Health Care for Women Int.

[CR4] Agoub M, Moussaoui D, Battas O (2005). Prevalence of postpartum depression in a Moroccan sample. Arch Women’s Mental Health.

[CR5] Al-Azri M, Al-Lawati I, Al-Kamyani R, Al-Kiyumi M, Al-Rawahi A, Davidson R, Al-Maniri A (2016). Prevalence and risk factors of antenatal depression among Omani women in a primary care setting. Sultan Qaboos Univ Med J.

[CR6] Al Dallal FH, Grant IN (2012). Postnatal depression among Bahraini women: prevalence of symptoms and psychosocial risk factors/Dépression postnatale chez des femmes bahreïnies: prévalence des symptômes et des facteurs de risque psychosociaux. East Mediterr Health J.

[CR7] Alharbi AA, Abdulghani HM (2014). Risk factors associated with postpartum depression in the Saudi population. Neuropsychiatr Dis Treat.

[CR8] Al Hinai FI, Al Hinai SS (2014). Prospective study on prevalence and risk factors of postpartum depression in Al-dakhliya governorate in Oman. Oman Med J.

[CR9] Barakat H (2005). The Arab family and the challenge of social transformation. Women Islam Crit Con Soc.

[CR10] Bener A (2013). Psychological distress among postpartum mothers of preterm infants and associated factors: a neglected public health problem. Rev Bras Psiquiatr.

[CR11] Bener A, Burgut FT, Ghuloum S, Sheikh J (2012). A study of postpartum depression in a fast developing country: prevalence and related factors. Int J Psychiatr Med.

[CR12] Bener A, Gerber LM, Sheikh J (2012). Prevalence of psychiatric disorders and associated risk factors in women during their postpartum period: a major public health problem and global comparison. Int J Womens Health.

[CR13] Burgut FT, Bener A, Ghuloum S, Sheikh J (2013). A study of postpartum depression and maternal risk factors in Qatar. J Psychosom Obstet Gynecol.

[CR14] Campbell C, Mannell J (2016). Conceptualising the agency of highly marginalised women: intimate partner violence in extreme settings. Global Public Health.

[CR15] Canadian Mental Health Association. (2017). Postpartum depression. http://www.cmha.ca/mental_health/postpartum-depression. Accessed on August 5, 2017

[CR16] Chaaya M, Campbell OMR, El Kak F, Shaar D, Harb H, Kaddour A (2002). Postpartum depression: prevalence and determinants in Lebanon. Arch Women’s Mental Health.

[CR17] Douki S, Zineb SB, Nacef F, Halbreich U (2007). Women’s mental health in the Muslim world: cultural, religious, and social issues. J Affect Disord.

[CR18] Eastwood JG, Phung H, Barnett B (2011). Postnatal depression and socio-demographic risk: factors associated with Edinburgh Depression Scale scores in a metropolitan area of New South Wales, Australia. Austr New Zealand J Psychiatry.

[CR19] Ehrlich M, Harville E, Xiong X, Buekens P, Pridjian G, Elkind-Hirsch K (2010). Loss of resources and hurricane experience as predictors of postpartum depression among women in southern Louisiana. J Women's Health.

[CR20] El-Khoury N, Karam EG, Melhem NM (1999). Depression et grossesse. Lebanese Med J.

[CR21] Eyadat Z (2013) Islamic Feminism: Roots, Development and Policies. Global Policy, 4(4), 359–368

[CR22] Fall A, Goulet L, Vézina M (2013). Comparative study of major depressive symptoms among pregnant women by employment status. SpringerPlus.

[CR23] Farr SL, Denk CE, Dahms EW, Dietz PM (2014). Evaluating universal education and screening for postpartum depression using population-based data. J Women's Health.

[CR24] Fuggle P, Glover L, Khan F, Haydon K (2002). Screening for postnatal depression in Bengali women: preliminary observations from using a translated version of the Edinburgh Postnatal Depression Scale (EPDS). J Reprod Infant Psychol.

[CR25] Glover V (2014). Maternal depression, anxiety and stress during pregnancy and child outcome; what needs to be done. Best Practice Res Clin Obs Gynaecol.

[CR26] Green K, Broome H, Mirabella J (2006). Postnatal depression among mothers in the United Arab Emirates: socio-cultural and physical factors. Psychol Health Med.

[CR27] Haghighat E (2013). Social status and change: the question of access to resources and women's empowerment in the Middle East and North Africa. J Int Women’s Stud.

[CR28] Haghighat E (2014). Establishing the connection between demographic and economic factors, and gender status in the Middle East: debunking the perception of Islam's undue influence. Int J Sociol Soc Policy.

[CR29] Hall KS, Kusunoki Y, Gatny H, Barber J (2014). The risk of unintended pregnancy among young women with mental health symptoms. Soc Sci Med.

[CR30] Hall KS, Kusunoki Y, Gatny H, Barber J (2015). Social discrimination, stress, and risk of unintended pregnancy among young women. J Adolesc Health.

[CR31] Hamdan A, Tamim H (2011). Psychosocial risk and protective factors for postpartum depression in the United Arab Emirates. Arch Women’s Mental Health.

[CR32] Hanmer L, Klugman J (2016) Exploring Women's Agency and Empowerment in Developing Countries: Where do we stand?. Feminist Economics, 22(1), 237–263

[CR33] Haque A, Namavar A, Breene K-A (2015). Prevalence and risk factors of postpartum depression in Middle Eastern/Arab women. J Muslim Mental Health.

[CR34] Higgins, J.P.T., & Green, S. (2008). Cochrane handbook for systematic reviews of interventions: Wiley Online Library

[CR35] Hill TD, Needham BL (2013). Rethinking gender and mental health: a critical analysis of three propositions. Soc Sci Med.

[CR36] James-Hawkins L, Peters C, VanderEnde K, Bardin L, & Yount KM (2016) Women’s agency and its relationship to current contraceptive use in lower-and middle-income countries: A systematic review of the literature. Global Public Health, 1–16. 10.1080/17441692.2016.1239270 10.1080/17441692.2016.1239270 27690750

[CR37] Kabeer N (1999). Resources, agency, achievements: reflections on the measurement of women’s empowerment. Dev Chang.

[CR38] Kabeer N (2016). Gender equality, economic growth, and women’s agency: the “endless variety” and “monotonous similarity” of patriarchal constraints. Fem Econ.

[CR39] Kabeer N, Assaad R, Darkwah A, Mahmud S, Sholkamy H, Tasneem S, Tsikata D (2013). Paid work, women’s empowerment, and inclusive growth: transforming the structures of constraint.

[CR40] Kandiyoti D (1991). Identity and its discontents: women and the nation. Millennium.

[CR41] Kandiyoti D (1988). Bargaining with patriarchy. Gender & Society.

[CR42] Khabour OF, Amarneh BH, Bani Hani EA, Lataifeh IM (2013). Associations between variations in TPH1, TPH2 and SLC6A4 genes and postpartum depression: a study in the Jordanian population. Balkan J Med Gen.

[CR43] Kronfol NM (2012). Access and barriers to health care delivery in Arab countries: a review/Accès et obstacles aux prestations de soins de santé dans les pays arabes: revue. East Mediterr Health J.

[CR44] Lancaster CA, Gold KJ, Flynn HA, Yoo H, Marcus SM, Davis MM (2010). Risk factors for depressive symptoms during pregnancy: a systematic review. Am J Obstet Gynecol.

[CR45] Leis JA, Heron J, Stuart EA, Mendelson T (2014). Associations between maternal mental health and child emotional and behavioral problems: does prenatal mental health matter?. J Abnorm Child Psychol.

[CR46] Lteif Y, Kesrouani A, Richa S (2005). Depressive syndromes during pregnancy: prevalence and risk factors. J de Gynecologie Obstetrique et biologie de la Reprod.

[CR47] Masmoudi J, Charfeddine F, Trabelsi S, Feki I, Ben AB, Guermazi M (2014). Postpartum depression: prevalence and risk factors. A prospective study concerning 302 Tunisian parturients. La Tunisie Medicale.

[CR48] Masmoudi J, Trabelsi S, Charfeddine F, Ben AB, Guermazzi M, Jaoua A (2008). Study of the prevalence of postpartum depression among 213 Tunisian parturients. Gynecolo Obstetrique & Fertilite.

[CR49] Masmoudi J, Trabelsi S, Charfeddine F, Ben AB, Guermazi M, Jaoua A (2010). Evaluation of affective temperaments in the postpartum depressive symptomatology. L'Encephale.

[CR50] McHichi Alami K, Kadri N, Berrada S (2006). Prevalence and psychosocial correlates of depressed mood during pregnancy and after childbirth in a Moroccan sample. Arch Women’s Mental Health.

[CR51] Miyake Y, Tanaka K, Arakawa M (2012). Employment, income, and education and prevalence of depressive symptoms during pregnancy: the Kyushu Okinawa Maternal and Child Health Study. BMC Psychiatry.

[CR52] Moh'd Yehia DB, Callister LC, Hamdan-Mansour A (2013). Prevalence and predictors of postpartum depression among Arabic Muslim Jordanian women serving in the military. J Perinat Neonatal Nurs.

[CR53] Mohammad KI, Gamble J, Creedy DK (2011). Prevalence and factors associated with the development of antenatal and postnatal depression among Jordanian women. Midwifery.

[CR54] Norwegian Knowledge Centre for the Health Services (2013). Effective Practice and Organisation of Care (EPOC) data collection form, EPOC resources for review authors. Oslo, Norway: Norwegian Knowledge Centre for the Health Services

[CR55] Parsons CE, Young KS, Rochat TJ, Kringelbach ML, Stein A (2012). Postnatal depression and its effects on child development: a review of evidence from low-and middle-income countries. Br Med Bull.

[CR56] Price A (2016). How national structures shape attitudes toward women’s right to employment in the Middle East. Int J Comp Sociol.

[CR57] Rashad H (2014). Health equity in the Arab world: the future we want. The Lancet, 383(9914), 286–287 10.1016/S0140-6736(13)62350-8 24452040

[CR58] Redshaw M, Henderson J (2016). Who is actually asked about their mental health in pregnancy and the postnatal period? Findings from a national survey. BMC Psychiatry.

[CR59] Rezaeian M (2010). Suicide among young Middle Eastern Muslim females. Crisis.

[CR60] Rubertsson C, Hellström J, Cross M, Sydsjö G (2014). Anxiety in early pregnancy: prevalence and contributing factors. Arch Women’s Mental Health.

[CR61] Satyanarayana VA, Lukose A, Srinivasan K (2011). Maternal mental health in pregnancy and child behavior. Indian J Psychiatry.

[CR62] Shaikh AK, Pearce B, & Yount, KM (2017) Effect of enabling resources and risk factors on the relationship between intimate partner violence and anxiety in ever-married women in Minya, Egypt. Journal of Family Violence, 32, 13–23. 10.1007/s10896-016-9848-5

[CR63] Scheyer K, Urizar GG (2016). Altered stress patterns and increased risk for postpartum depression among low-income pregnant women. Archi Women's Mental Health.

[CR64] Sewilam AM, Watson AM, Kassem AM, Clifton S, McDonald MC, Lipski R, Deshpande S, Mansour H, Nimgaonkar VL (2015). Suggested avenues to reduce the stigma of mental illness in the Middle East. Int J Soc Psychiatry.

[CR65] Von Elm E, Altman DG, Egger M, Pocock SJ, Gøtzsche PC, Vandenbroucke JP (2007). The Strengthening the Reporting of Observational Studies in Epidemiology (STROBE) statement: guidelines for reporting observational studies. Prev Med.

[CR66] World Health Organization. (n.d.). Maternal mental health. Accessed on 10/28/2016 at: http://www.who.int/mental_health/maternal-child/maternal_mental_health/en/

[CR67] World Health Organization, United Nations Population Fund, & Key Centre for Women’s Health in Society. (2009). Mental health aspects of women’s reproductive health: a global review of the literature: World Health Organization

[CR68] Yelland J, Sutherland G, Brown SJ (2010). Postpartum anxiety, depression and social health: findings from a population-based survey of Australian women. BMC Public Health.

[CR69] Yount KM, Smith SM (2012). Gender and postpartum depression in Arab Middle Eastern women. Women’s Studies International Forum, 35, 187–193. 10.1016/j.wsif.2012.03.017

[CR70] Yount KM, Dijkerman S, Zureick-Brown S, & VanderEnde, K.E. (2014). Women's empowerment and generalized anxiety in Minya, Egypt. Social Science & Medicine, 106, 185–193. 10.1016/j.socscimed.2014.01.022 10.1016/j.socscimed.2014.01.022 24576646

